# High-throughput peptide quantification using mTRAQ reagent triplex

**DOI:** 10.1186/1471-2105-12-S1-S46

**Published:** 2011-02-15

**Authors:** Joo Young Yoon, Jeonghun Yeom, Heebum Lee, Kyutae Kim, Seungjin Na, Kunsoo Park, Eunok Paek, Cheolju Lee

**Affiliations:** 1 School of Computer Science and Engineering, Seoul National University, Seoul, 151-742, Korea; 2 Life Sciences Division, Korea Institute of Science and Technology, Seoul, 136-791, Korea; 3 Department of Biomolecular Science, University of Science and Technology, Daejeon, 305-333, Korea; 4 Department of Mechanical and Information Engineering, University of Seoul, Seoul, 130-743, Korea

## Abstract

**Background:**

Protein quantification is an essential step in many proteomics experiments. A number of labeling approaches have been proposed and adopted in mass spectrometry (MS) based relative quantification. The mTRAQ, one of the stable isotope labeling methods, is amine-specific and available in triplex format, so that the sample throughput could be doubled when compared with duplex reagents.

**Methods and results:**

Here we propose a novel data analysis algorithm for peptide quantification in triplex mTRAQ experiments. It improved the accuracy of quantification in two features. First, it identified and separated triplex isotopic clusters of a peptide in each full MS scan. We designed a schematic model of triplex overlapping isotopic clusters, and separated triplex isotopic clusters by solving cubic equations, which are deduced from the schematic model. Second, it automatically determined the elution areas of peptides. Some peptides have similar atomic masses and elution times, so their elution areas can have overlaps. Our algorithm successfully identified the overlaps and found accurate elution areas. We validated our algorithm using standard protein mixture experiments.

**Conclusions:**

We showed that our algorithm was able to accurately quantify peptides in triplex mTRAQ experiments. Its software implementation is compatible with Trans-Proteomic Pipeline (TPP), and thus enables high-throughput analysis of proteomics data.

## Background

Introduction of mass spectrometry (MS) provides massive biological information of proteins for both qualitative and quantitative analysis [1]. Recently, quantitative analyses have become of particular interest in proteomics research [2]. To determine the expressional differences of proteins across samples representing different physiological or disease states, various experimental approaches have been developed: spectral counting, stable isotope labeling, and label-free quantification [3].

Stable isotope labeling is one of popular methods for protein quantification. Peptides of two or more samples are differently labeled using stable isotopes to introduce mass shifts. Then they are experimented within a single LC/MS run, so that the sample throughput could be multiplied when compared with that of label-free quantification. There are various labeling techniques: ICAT [4], SILAC [5], ^18^O labelling [6], iTRAQ [7], mTRAQ [8], and so on. Numerous computational tools for the stable isotope labeling have also been developed, including XPRESS [9], ASAPRatio [10], STEM [11], ZoomQuant [12], MSInspect [13], Multi-Q [14], Q3 [15], VIPER [16], MaxQuant [17], Census [18], and IEMM [19].

In this paper, we focus on the isotope label mTRAQ, which is a nonisobaric variant of the iTRAQ and was originally designed for multiple reaction monitoring (MRM) [20]. The mTRAQ labels were first designed in two chemically identical versions. The heavy-label is identical to the iTRAQ 117 label and its mass is 145 Da. The light-label is chemically identical to the heavy-label, but it has no 13C or 15N, so its mass is 141 Da. They are labeled at lysine residue and N-terminal. We verified that the mTRAQ is a powerful isotope label for MS-based relative quantification [8], and developed a new algorithm to improve the accuracy of peptide quantification in mTRAQ labeling based MS experiments [21]. Recently, the mTRAQ has become available in triplex format, where the label with 149 Da is added.

One of the major obstacles to accurate peptide quantification is the overlap of isotopic clusters. There are two types of overlap problems, one is the overlap between differently labeled peptides, and the other is the overlap between chemically different peptides. The former can happen when the mass difference between labels is very small. In mTRAQ experiments, the mass difference between differently labeled peptides is 4 Da if the original peptide has no lysine, so it is important to separate their isotopic clusters correctly. The latter could be found in all kinds of MS-based experiments. For peptide quantification, most of the times we are interested in relative quantification of peptides whose amino acid sequences are known. When we know the sequences of peptides of interest, there are better chances to recognize the overlaps from differential labeling by comparing them to the theoretical isotopic distributions.

In this manuscript, we present a new data analysis algorithm for peptide quantification in triplex mTRAQ experiments. It is an extension of the algorithm for duplex mTRAQ experiments [21]. We identify isotopic clusters of triplex labeled peptides and separate their intensities using cubic equation modelling when there are overlaps. We also designed an automatic determination algorithm for the elution area of peptides, which could recognize the overlap between chemically different peptides. We demonstrate the performance of our algorithm using standard protein mixture experiments.

## Materials and methods

### Preparation of standard samples

Three kinds of standard protein mixtures (Std1, Std2, and Std3) were prepared for mTRAQ quantification testing by mixing 7 bovine proteins. Each mixture consisted of alpha-lactalbumin (LALBA), beta-casein (CSN2), serotransferrin (TF), alpha-S1-casein (CSN1S1), alpha-S2-casein (CSN1S1), cytochrome c (CYCS) and beta-lactoglobulin (LGB) in 50 mM Tris pH 8.0 at different amounts as summarized in Table 1.

**Table 1:** Standard protein mixtures

Protein	Std1 (μg)	Std2 (μg)	Std3 (μg)
alpha-lactalbumin (LALBA)	5	5	5
beta-casein (CSN2)	5	10	1
Serotransferrin (TF)	10	1	3
alpha-S1-casein (CSN1S1)	1	1	3
alpha-S2-casein (CSN1S2)	1	1	3
cytochrome c (CYCS)	3	3	1
beta-lactoglobulin (LGB)	1	5	10
Total	26	26	26

### mTRAQ labeling

The standard protein mixtures were labeled with mTRAQ^TM^ reagent (AB Sciex, Foster City, CA, USA) as described in [8] and [21]. Proteins were reduced with 50 mM tris (2-carboxyethyl) phosphine (Thermo Fisher Scientific, Rockford, IL, USA) for 1 hr at 60 °C, treated with 200 mM methyl methanethiosulfonate (MMTS; Tokyo Chemical Industry, Tokyo, Japan) for 10 min at 25 °C, and then diluted 10 fold with 50 mM Tris (pH 8.0), and digested with sequencing-grade trypsin (Promega, Madison, WI, U.S.A.) at 37 °C overnight at the protein:trypsin molar ratio of 40:1. Tryptic digests were desalted with C18 solid-phase extraction cartridge and dried in vacuo. The dried samples were reconstituted in 500 mM triethylammonium bicarbonate (Sigma-Aldrich St Louis, MO, USA) and incubated with appropriate mTRAQ reagents at 25 °C for 1 hr. For the Set1 experiment, Std1 was labeled with mTRAQ® ∆0 (Light), Std2 with mTRAQ® ∆4 (Medium), and Std3 with mTRAQ® ∆8 (Heavy). For the Set2 experiment, Std1 was labeled with Heavy reagent, Std2 with Medium, and Std3 with Light (Table 1). After the labeling reaction, samples were dried in vacuo, redissolved in 0.1% trifluoroacetic acid, mixed equally, desalted with a mixed-mode strong cation-exchange (MCX) cartridge and dried again.

### Mass spectrometric analyses of mTRAQ labeled samples

Labeled sample mixtures were reconstituted in 0.4% acetic acid and an aliquot (~1 μg) was injected to a reversed-phase Magic C18aq column (15 cm x 75 μm) on an Eksigent multi-dimensional liquid chromatography (MDLC) system at the flow rate of 300 nL/min. The column was equilibrated with 95% buffer A (0.1% formic acid in H_2_O) + 5 % buffer B (0.1% formic acid in acetonitrile) prior to use. The peptides were eluted with a linear gradient of 10 to 40% Buffer B over 40 min.

The high performance liquid chromatography (HPLC) system was coupled to a linear trap quadrupole (LTQ) XL-Orbitrap mass spectrometer (Thermo Scientific, San Jose, CA, U.S.A.). The spray voltage was set to 1.9 kV, and the temperature of the heated capillary was set to 250 °C. Survey full-scan MS spectra (*m*/*z* 300–2,000) were acquired in the Orbitrap with 1 microscan and a resolution of 100,000 allowing the preview mode for precursor selection and charge-state determination. MS/MS spectra of the five most intense ions from the preview survey scan were acquired in the ion-trap concurrently with full-scan acquisition in the Orbitrap with the following options: isolation width, ±10 ppm; normalized collision energy, 35%; dynamic exclusion duration, 30 sec. Precursors with unmatched and single charge states were discarded during data dependant acquisition. Data were acquired using the Xcalibur software v2.0.7 (Thermo Scientific).

### Database searching of MS/MS data for peptide identification

The data files collected on the mass spectrometer (.raw) were converted to MGF format by use of Trans-Proteomic Pipeline (TPP, version 4.3 JETSTREAM rev 1, http://www.proteomecenter.org), which is an open source proteomics analysis tool. The data were then searched using MASCOT [22] (version 2.2.06) against a compound database consisting of the International Protein Index (IPI, European Bioinformatics Institute, http://www.ebi.ac.uk/IPI) bovine database (version 3.42) and IPI human database (version 3.57) totaling 107,511 protein entries, allowing the options of trypsin, ±0.5 Da mass tolerance for fragment ion, ±15 ppm mass tolerance for precursor ion, variable modifications of mTRAQ Light (+140.095 Da), mTRAQ Medium (+144.1021 Da) and mTRAQ Heavy (+148.1092 Da) on peptide N-terminus and Lys residue. A fixed modification of MMTS (+45.9877 Da) on Cys residue and a variable modification of Met oxidation (+15.9949) were also allowed. TPP was used for the validation of database search results. Peptides with TPP peptide probability greater 0.9 and MASCOT E-value less than 0.01 were used for further quantification analysis.

### Overview of the algorithm

Our algorithm is designed to be executed within TPP. For each LC/MS experiment, TPP generates a pepXML file which contains a list of peptides with sequences, tandem scans, charges, and modifications. Our algorithm calculates medium to light (M/L) and heavy to light (H/L) ratios of peptides in pepXML files and produces new pepXML files that can be used for further analysis. For each peptide, our algorithm first determines its elution area. It then identifies triplex isotopic clusters and calculates M/L and H/L ratios for each MS scan contained in the elution area. Finally, each of the set of M/L and H/L ratios is integrated based on linear regression.

### Model of overlapping isotopic clusters

**Figure 1:**
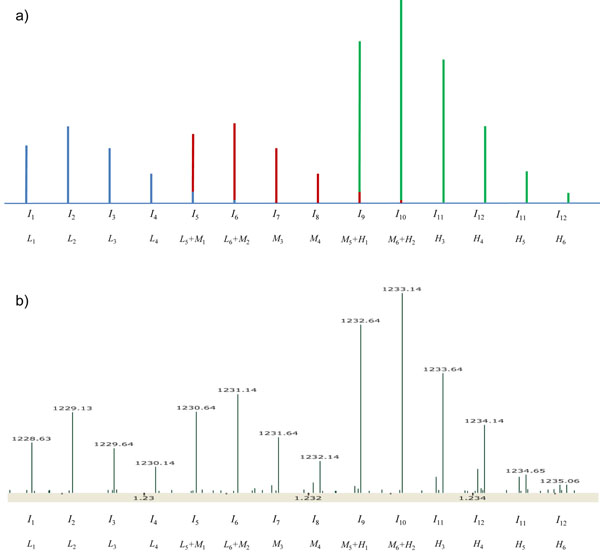
**Examples of overlapping triplex isotopic clusters**. (a) Schematic model of overlapping triplex isotopic clusters. Blue, red, and green lines represent isotopic peaks of light-, medium-, and heavy-labeled peptides, respectively. (b) Experimental overlapping isotopic clusters of ‘EPMIGVNQELAYFYPELFR’. We can observe that the first isotopic peaks of medium- and heavy-labeled peptides are clearly higher than the corresponding third peaks, and it is evidence for the existence of overlaps.

We made a schematic model of overlapping triplex isotopic clusters, which is an extension of the model in our previous work (Figure 1) [21]. We assumed that an isotopic cluster of a peptide has 8 or less peaks. Such an assumption is reasonable for peptides whose masses are less than 4000 Da because the relative intensity of the ninth peak in the theoretical distribution of a 4000 Da peptide is only 0.56% according to an averagine model [23]. Under this assumption, an overlap exists only if a peptide has no lysine. In this case, the mass difference between labeled peptides is 4 Da, thus the intensity of the *k*th peak *I_k_* is given as follows:(1)

where *n* is the number of peaks in the isotopic distribution of a peptide, *L_k_*, *M_k_*, and *H_k_* are the intensities of the *k*th peaks of the isotopic distributions of the light, medium, and heavy-labeled peptides, respectively.

Let *α* be the M/L ratio and *β* be the H/L ratio. For 1 ≤ *k* ≤ 4, it is easy to show(2)

from equation (1). Using equation (2), we induced three equations(3)(4)(5)

From equations (4) and (5), we obtain a cubic equation for *β*:(6)

Solving equation (6), we obtain up to three candidate values for *β*. Then, by substituting the candidates into equation (3) and solving it, we obtain up to two candidate values for *α*. (Substituting candidates for *β* into equation (4) may lead to an abnormal *α* value because *I_k_*_+12_ could possibly be very small and inaccurate in its value. Substituting into Equation (5) could also be problematic because a small value of *β* could cause an inaccurate *α* value.) To select the most accurate ratio pair, we define an error function as follows:(7)

where *T_k_* is the intensity of the *k*th peak of the theoretical isotopic distribution of the peptide. (The EMASS algorithm was used to calculate *T_k_* values [24].) The error value should be very small for the correct ratio pair because *L_k_*_+4_/*L_k_*, *M_k_*_+4_/*M_k_*, and *H_k_*_+4_/*H_k_* are theoretically the same as *T_k_*_+4_/*T_k_*. Therefore, we calculated the error value for each candidate pair and select the pair with the lowest error value. After all pairs for 1 ≤ *k* ≤ 4 are selected, we can calculate the M/L ratio  and the H/L ratio .

### Determination of the elution areas of peptides

In most LC/MS experiments, tandem MS scans are acquired using dynamic exclusion (DE). For each MS/MS scan, therefore, we know only one MS scan where the identified peptide is eluted. We need to determine the elution area of the peptide as it is eluted over a period of time. However, some peptides have similar atomic masses and elution times, so their elution areas can have overlaps. A naive approach such as using a fixed range (e.g. within ±30s from the tandem scan of peptides) has a risk of including incorrect MS scans where other peptides are overlapped. Therefore, it is very important to determine accurate elution areas of the peptides for accurate relative quantification.

We assume that the distribution of peptide elution time can be approximated as a normal distribution. Because of noise and overlap of peptides, MS scans with low intensities at both ends of the elution area may not be trusted. If we use only MS scans with high total ion current while modeling the elution profile as a normal distribution, the mean μ of the normal distribution can be approximated, but the variance σ^2^ can’t. Instead, we use the full width at half maximum (FWHM) to induce σ^2^. From the probability density function of the normal distribution, we deduce  and obtain σ^2^ = FWHM^2^/8 ln 2.

**Figure 2:**
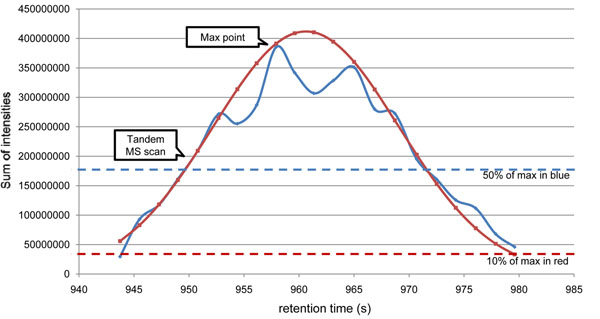
**Elution area approximation to normal distribution.** Elution area approximation for ‘HPIKHQGLPQEVLNENLLR’. The blue line represents the sum of intensities of the peptide over the elution area and the red line is an approximated normal distribution. From the given elution time (951.423 s), where tandem MS scan was acquired, we first found the maximum point of elution area (957.93 s). Then we extended the area until the sum of intensities is below 50% of that of the maximum point and obtained μ = 960.63 and FWHM = 19.9. Finally, we used the area with higher intensities than 10% of the maximum intensity of the approximated normal distribution.

When a peptide identification and the associated tandem MS scan is given, our algorithm first finds the maximum point of the peptide’s elution profile. For each MS scan within ±30s range from the given tandem scan, it identifies triplex isotopic clusters and calculates the sum of intensities. (Details are explained in the next section.) The MS scan whose sum of intensities is the highest is selected as the maximum point of the elution area. Then it extends the elution area while the sum of intensities of MS scan is above a half of that of the maximum point. The length of the extended area is used as FWHM and weighted average time of scans in the extended area is used as μ. The area with higher intensities than 10% of the maximum intensity in the normal distribution (from  to ) is used as the elution area of a peptide. An example for approximation to normal distribution is shown in Figure 2.

Our algorithm calculates M/L and H/L ratios for all MS scans in the elution area. Then, each of the set of M/L and H/L ratios is integrated by linear regression using the form “*y* = *cx*”. The intensities of peaks are split into the intensity of light-, medium-, and heavy-labeled peptide. We estimate *c* using the set of intensities of light-labeled peptides as *x_i_*’s, and the set of intensities of medium- and heavy-labeled peptides as *y_i_*’s for M/L and H/L ratios, respectively.

### Identification and validation of triplex isotopic clusters

**Figure 3:**
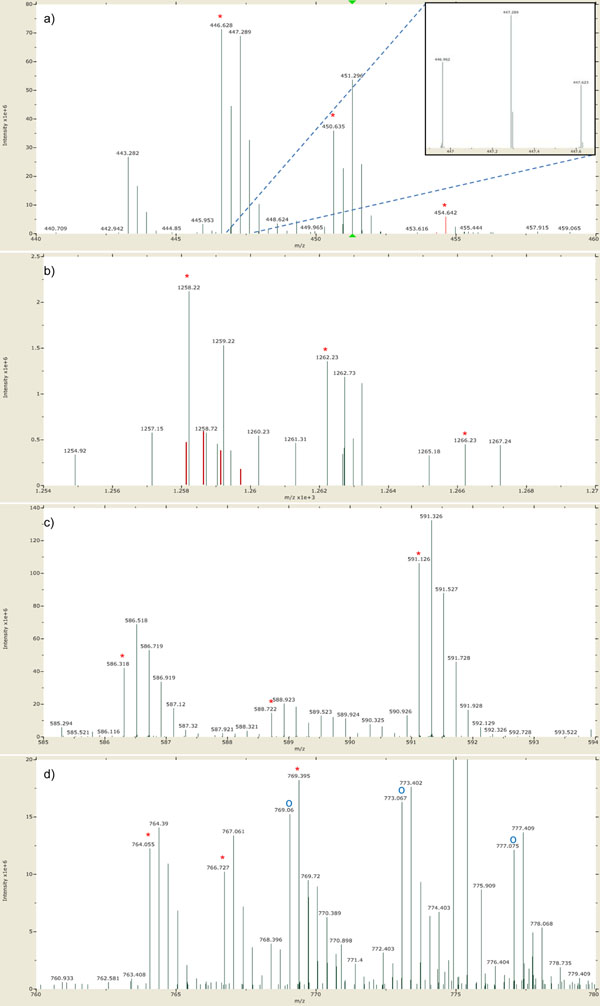
**Four types of overlaps between chemically different peptides**. The red stars represent the monoisotopic peaks of triplex isotopic clusters of a target peptide. (a) Two isotopic clusters are overlapped, but no isotopic peak is shared. (b) An MS scan in the elution area of ‘HPIKHQGLPQEVLNENLLR’ of which expected ratio is 3:1:1. The red lines represent its theoretical isotopic distribution. Since an isotopic cluster with a different charge value is overlapped with the light-labeled isotopic cluster, the LSQ value becomes significantly high, so we discard this MS scan during the quantification of the target peptide. (c) An MS scan in the elution area of ‘TVGGKEDVIWELLNHAQEHFGK’ of which expected ratio is 3:1:10. An isotopic cluster with the same charge and a higher mass is overlapped with the medium-labeled isotopic cluster. Since the fifth peak increases the LSQ value, only the first to the fourth peaks are used to quantify. (d) An MS scan in the elution area of ‘GITWGEETLMEYLENPK’ of which expected ratio is 3:3:1. An isotopic cluster with the same charge and 1 Da smaller mass is overlapped with the heavy-labeled isotopic cluster. Since it is difficult to separate these overlapping isotopic clusters accurately, we discard this MS scan during the quantification.

For each MS scan in the elution area, our algorithm identifies isotopic clusters of a target peptide. Let *MZ_k_* be the *m*/*z* of the *k*th peak of an isotopic cluster, then we can calculate three *MZ*_1_’s corresponding to triplex isotopic clusters from the given sequence, charge *z*, and modification. Our algorithm first finds the monoisotopic peak of each isotopic cluster from *MZ*_1_ within 10 ppm error tolerance. Then, it finds subsequent isotopic peaks from *MZ_k_ = MZ_k-_*_1_*+* 1.00235/*z* within 10 ppm error tolerance. The *k*th peak is inserted to the isotopic cluster only if the peak improves the least squares fit value (LSQ). If the LSQ between the theoretical distribution of the peptide and the isotopic cluster without the *k*th peak is lower than that with the *k*th peak, the *k*th peak is discarded and the algorithm does not look for any more peaks. If there are two or more candidate peaks for the *k*th peak, the peak with the lowest LSQ is selected. For example, there are two candidates for the third isotopic peak of the light-labeled isotopic cluster of the target peptide and the smaller peak is selected in Figure 3a.

After identification of triplex isotopic clusters of a target peptide, we check them and discard the current MS scan if they are doubtful according to the following criteria. First, we check whether the overall shape of each isotopic cluster resembles that of a theoretical isotopic distribution. At least the LSQ of the most abundant isotopic cluster must be below a threshold (e.g. 0.2). The LSQ of the others should also be below the threshold unless their sums of intensities are lower than a half of that of the most abundant isotopic cluster. (If an isotopic cluster has low abundance, its shape could be abnormal because it may be interfered by chemical noise and other peptides.) Second, we check whether the identified isotopic cluster is overlapped with another peptide. Four types of overlaps are shown in Figure 3. There is no problem if no isotopic peak is shared by two isotopic clusters (Figure 3a). If an isotopic cluster with a different charge value is overlapped, the LSQ of the identified isotopic cluster should be significantly high, so we can discard the current MS scan (Figure 3b). If an isotopic cluster with the same charge and a higher mass is overlapped, shared isotopic peaks could not be inserted to the isotopic cluster of the target peptide because it increases the LSQ of the isotopic cluster (Figure 3c). Only the case in which an isotopic cluster with the same charge and a lower mass is overlapped needs additional filtering (Figure 3d). We can easily detect these overlaps by considering previous peaks, but we can’t separate overlapping isotopic clusters in this case because they look like one isotopic cluster. Therefore, we discard the current MS scan if at least one isotopic cluster of a target peptide could be identified as an isotopic cluster with the same charge and a lower mass.

## Results and discussion

### Application to 7-standard protein data mixed with known ratios

We analyzed two datasets in which seven standard proteins were mixed in different ratios. For the Set1 experiment, Std1 was labeled with light, Std2 with medium, and Std3 with heavy. For the Set2 experiment, Std1 was labeled with heavy, Std2 with medium, and Std3 with light. The expected ratios for each experiment are shown in Table 2.

**Table 2:** Expected ratios and computed ratios for seven proteins in standard mixtures

(a) Set1 experiment							
Protein	Number of MS/MS	M/L			H/L		

		Expected ratio	Our ratio	Standard deviation	Expected ratio	Our ratio	Standard deviation

LALBA	17	1	1.074858	0.085456	1	0.695681	0.072863
CSN2	3	2	4.847636	0.093352	0.2	0.184493	0.045277
TF	76	0.1	0.098397	0.186558	0.3	0.160631	0.141608
CSN1S1	10	1	1.264178	0.10274	3	1.948002	0.085701
CSN1S2	4	1	2.419368	0.133448	3	1.644656	0.088651
CYCS	15	1	0.846116	0.068538	0.3	0.34976	0.123581
LGB	22	5	5.138141	0.181286	10	8.143161	0.174779

(b) Set2 experiment							

Protein	Number of MS/MS	M/L			H/L		

		Expected ratio	Our ratio	Standard deviation	Expected ratio	Our ratio	Standard deviation

LALBA	18	1	1.010655	0.098869	1	0.487596	0.076048
CSN2	3	10	13.64935	0.112667	5	1.517403	0.112521
TF	64	0.33	0.43843	0.166834	3.3	2.829079	0.108205
CSN1S1	13	0.33	0.471544	0.071577	0.33	0.223681	0.084549
CSN1S2	10	0.33	0.984124	0.042867	0.33	0.274309	0.083823
CYCS	13	3	1.83768	0.049094	3	1.417869	0.083364
LGB	18	0.5	0.436385	0.049273	0.1	0.050817	0.220646

We first calculated the averages and standard deviations of log_10_(H/L) values of peptides. Then, we transformed the averages into H/L scale to compare them to expected ratios.

**Figure 4:**
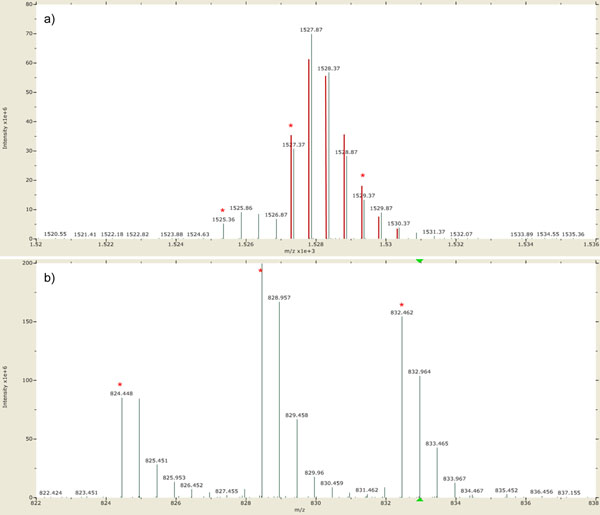
**Manual inspection for the peptides whose computed ratios are different from the expected ratio**. a) The most abundant isotopic clusters for ‘DMPIQAFLLYQEPVLGPVRGPFPIIV’. The red lines represent its theoretical isotopic distribution. The expected ratio is 5:10:1, and our algorithm computed 5.612575 as M/L and 0.163601 as H/L for this peptide. (b) The most abundant isotopic clusters for ‘ALNEINQFYQK’. The expected ratio is 1:1:3, and our algorithm computed 2.074808 as M/L and 1.48508 as H/L. It is clear that our ratios are more suitable than the expected ratios for these isotopic clusters.

**Figure 5:**
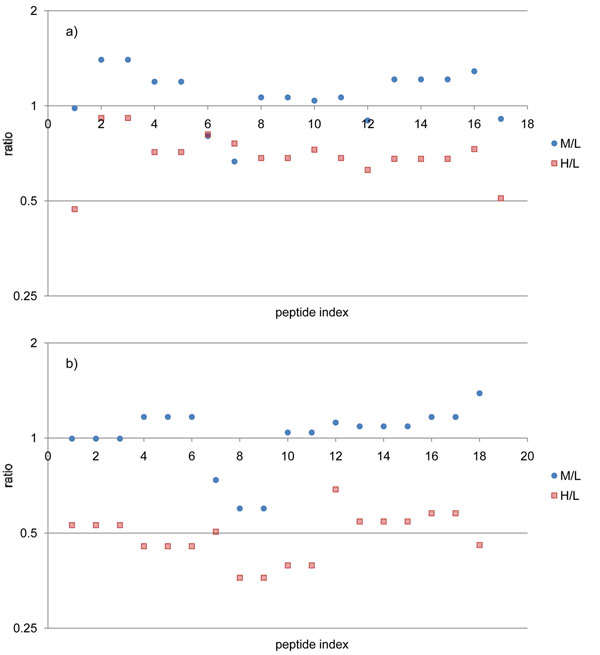
**Distribution of ratios of peptides for LALBA**. a) Set1 experiment, (b) Set2 experiment

After validation, we obtained 147 MS/MS scans from Set1 and 139 MS/MS scans from Set2, resulting in 168 unique peptides in total. We calculated M/L and H/L ratios of the peptides and classified them according to the proteins. Then we calculated the averages of ratios in individual cases and compared them to the expected ratios (Table 2). The M/L ratios were generally similar to the expected ratios except CSN2 and CSN1S2, whose ratios were somewhat higher than expected ratios. Most H/L ratios were somewhat lower than the expected ratios, but their standard deviations are meaningfully small. We manually inspected the isotopic clusters of these peptides and concluded that the computed ratios are certainly correct despite their discrepancy from the expected ratios. Some examples of these cases are shown in Figure 4. In spite of our effort to label the samples and to mix them accurately, the mixed ratios of samples may deviate from the expected ratio because of different labeling efficiencies between the labels, and experimental errors such as unequal mixing. Average ratio and standard deviation are the two parameters that determine the accuracy of our quantification analysis. Unlike the average ratio that is very sensitive to such errors, standard deviation is more inert because the ratios originated from peptides of the same protein should be identical in theory. Therefore, the low standard deviations give strong evidence that our computed ratios were accurately determined. Figure 5 shows the distribution of ratios for LALBA. Each of M/L and H/L ratios represent similar values. The distributions of ratios for the other proteins are given in Supplementary Figure S1 – in Additional file 1.

### Separation of overlapping triplex isotopic clusters

**Figure 6:**
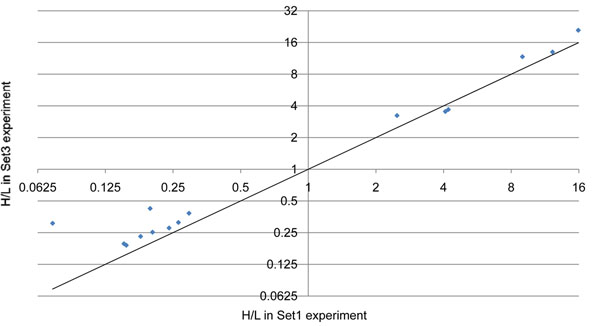
**Comparison of H/L ratios between Set1 experiment (including medium label) and Set3 experiment (excluding medium label)**. Each point represents a unique peptide that has no lysine. The solid line represents *y*=*x*. For Set1 experiment, Std1 with light, Std2 with medium, and Std3 with heavy label were mixed. For Set3 experiment, Std1 with light and Std3 with heavy label were mixed. The H/L ratios in the Set1 experiment were very close to the H/L ratios in the Set3 experiment in spite of the interference by the medium-labeled peptides.

To verify the robustness of our method for the overlap of triplex isotopic clusters, we prepared Set3 experiment, in which Std1 with light and Std3 with heavy were mixed. In the Set3 experiment, two isotopic clusters originated from the same peptide have no overlap and their relative ratio can be computed accurately even though the peptide has no lysine. Therefore, we can show the robustness of our method by comparing the H/L ratios from the Set1 experiment to those from the Set3 experiment. Fifteen unique peptides were identified in both experiments and their H/L ratios are shown in Figure 6. The H/L ratios in the Set1 experiment were very close to the H/L ratios in the Set3 experiment in spite of the interference of the medium-labeled peptides. The relative ratios of two H/L ratios ranged from 0.868378 to 1.315178, except two peptides from CSN2 whose expected L:M:H ratios in the Set1 experiment were 5:10:1. The H/L ratios of the peptides of CNS2 were somewhat lower in the Set1 experiment than in the Set3 experiment because the medium-labeled isotopic cluster was much larger than the heavy-labeled isotopic cluster and influenced it (Figure 4a).

### Cause of low abundance of heavy-labeled peptides

Std1 and Std3 were labeled with light and heavy mTRAQ labels, respectively, in Set1 Experiment and vice-versa in Set2 Experiment. The calculated H/L ratios were lower than the estimated values in both cases, which exclude the possibility of under-digestion of some of the standard mixtures compared to the others. If then, we would expect reversed H/L ratios between the two experimental sets. It becomes even more evident if we consider the MS/MS search results in which only one out of 168 validated peptides was identified as partially labeled.

The most probable explanation at this point is low labeling efficiency of the heavy reagent. If we assume that the M/L ratios are correct, we can approximate the H/L ratios in Set1 experiment using M/L ratios in Set2 experiment. Similarly, we can approximate the H/L ratios in Set2 experiment using M/L ratios in Set1 experiment. We compared them with the computed H/L ratios and observed that the computed H/L ratios are consistently 50~70% of the approximated H/L ratios except for the cases of CYCS in Set1 experiment (Table 3). This result shows the possibility that the heavy reagent had low labeling efficiency.

**Table 3:** Comparison between approximated H/L ratios and computed H/L ratios

Protein	Set1			Set2		

	approximated H/L	computed H/L	Computed /approximated	approximated H/L	computed H/L	Computed /approximated
LALBA	1.063526	0.695681	0.654127	0.940268	0.487596	0.518571
CSN2	0.355155	0.184493	0.519472	2.815671	1.517403	0.538913
TF	0.22443	0.160631	0.715728	4.455725	2.829079	0.634931
CSN1S1	2.680934	1.948002	0.726613	0.373004	0.223681	0.599674
CSN1S2	2.458398	1.644656	0.668995	0.406769	0.274309	0.674361
CYCS	0.460426	0.068538	0.148858	2.171901	1.417869	0.652824
LGB	11.77433	8.143161	0.691603	0.084931	0.050817	0.598336

The origin can also be explained, though in part, by isotope impurity of heavy label. Upon closer inspection of MS spectra of the identified peptides, a peak 1 Da smaller than the monoisotopic peak of heavy label was frequently found (Supplementary Figure S2 in Additional file 1). It was reported that iTRAQ reagents contain trace levels of isotopic impurities [25]. Since mTRAQ shares the same chemical structure with iTRAQ, we expect that the same problem will happen in mTRAQ data analysis.

In real experiments where quantification of complex proteome is needed, one can add a known standard at the ratio of 1:1:1, and use the calculated ratio of the standard as a correction factor. For example, if the calculated ratio of LALBA in the current study is used as a correction factor, the ratios of other proteins become closer to the expected ratios.

## Conclusions

We have developed a new data analysis algorithm for peptide quantification in triplex mTRAQ experiments. It can calculate the ratios of peptides accurately by separating overlapping triplex isotopic clusters based on the arithmetic models of isotope overlap and an automatic determination for the elution area of peptides. When used within the TPP pipeline, it can easily analyze high-throughput proteomics data.

## Competing interests

The authors declare no competing financial interests.

## Authors' contributions

JYY, KP, EP and CL conceived the research. JYY and HL implemented the program. JY and KK prepared experimental datasets. SN identified the sequence of peptides by database searching. JYY and HL analyzed the result of the program. JYY, KP, EP, JY and CL wrote and revised the manuscript. All authors read and approved the final manuscript.

## Supplementary Material

Additional file 1 **Supplementary Material** Supplementary Figures. Click here for file
