# Supplementary material for: High-throughput peptide quantification using mTRAQ reagent triplex
Source: BMC Bioinformatics. 2011 Feb 15;12(Suppl 1):S46. doi: 10.1186/1471-2105-12-S1-S46 (PMC3044303; doi:10.1186/1471-2105-12-S1-S46)
Supplement: Additional file 1 — Supplementary Material Supplementary Figures. [file 1471-2105-12-S1-S46-S1.docx]

## Supplementary Figure 1 - Distribution of ratios of peptides for six proteins

1. Distribution of ratios of peptides for CSN2 in Set1 experiment
2. Distribution of ratios of peptides for CSN2 in Set2 experiment
3. Distribution of ratios of peptides for TF in Set1 experiment
4. Distribution of ratios of peptides for TF in Set2 experiment
5. Distribution of ratios of peptides for CSN1S1 in Set1 experiment
6. Distribution of ratios of peptides for CSN1S1 in Set2 experiment
7. Distribution of ratios of peptides for CSN1S2 in Set1 experiment
8. Distribution of ratios of peptides for CSN1S2 in Set2 experiment
9. Distribution of ratios of peptides for CYCS in Set1 experiment
10. Distribution of ratios of peptides for CYCS in Set2 experiment
11. Distribution of ratios of peptides for LGB in Set1 experiment
12. Distribution of ratios of peptides for LGB in Set2 experiment

## Supplementary Figure 2 - Isotope impurity of heavy label

1. The most abundant isotopic clusters for ‘VGINYWLAHK’
2. The most abundant isotopic clusters for ‘LDQWLCEKL’

The red stars represent the monoisotopic peaks of the triplex isotopic clusters of the target peptide and the blue circles represent the peaks 1 Da smaller than the monoisotopic peak.
